# Cognitive Impairment in People Living with HIV and the Impact of Mood: Results from a Cross-Sectional Study

**DOI:** 10.3390/jcm13061631

**Published:** 2024-03-13

**Authors:** Francesco Salis, Maristella Belfiori, Alice Bellisai, Eleonora Bernardini, Michele Murtas, Rossella Piras, Silvia Serreli, Francesco Ortu, Paola Piano, Stefano Del Giacco, Antonella Mandas

**Affiliations:** 1 Department of Medical Sciences and Public Health, University of Cagliari, SS 554 bivio Sestu, 09042 Cagliari, Italyalicebellisai7@gmail.com (A.B.); eleb94@live.it (E.B.); mod85mike@gmail.com (M.M.); pirasrossellaa@gmail.com (R.P.); silviaserreli86@gmail.com (S.S.); delgiacco@unica.it (S.D.G.); amandas@unica.it (A.M.); 2 Department of Biomedical Sciences, University of Cagliari, 09042 Cagliari, Italy; 3 University Hospital “Azienda Ospedaliero-Universitaria” of Cagliari, 09042 Cagliari, Italy; fortu@aoucagliari.it (F.O.); ppiano@aoucagliari.it (P.P.)

**Keywords:** HIV, cognitive assessment, Montreal Cognitive Assessment (MoCA), HIV-associated neurocognitive disorder (HAND)

## Abstract

**Background**: Human Immunodeficiency Virus (HIV) infection represents a significant public health concern and, consequently, the incidence of HIV-Associated Neurocognitive Disorder (HAND) has grown over the years. The present study aims to assess HAND with the Montreal Cognitive Assessment (MoCA) in People Living With HIV/AIDS (PLWHA) to find significant associations with cognitive impairment. **Methods**: The study included 210 PLWHA, aged from 30 to 81 years, of whom, 137 (65.2%) were males. They were assessed at the Immunology Service of the University Hospital of Monserrato, Cagliari, Italy, between November 2022 and April 2023. **Results**: The sample showed an overall optimal response to antiretroviral therapy, as shown by the excellent levels of CD4+ lymphocytes and HIV RNA copies. A sum of 115 subjects (54.8%) were considered cognitively impaired and the multivariate analysis demonstrated that it was independently associated with duration of infection (OR: 0.96), age (OR: 1.12), alanine aminotransferase (ALT) (OR: 1.02), and depression (OR: 1.33). By dichotomizing the variables, the significance of the association was confirmed for age (65-year threshold) (χ^2^: 5.142, *p* = 0.0233) and depression (χ^2^: 7.834, *p* = 0.0051). **Conclusions**: Our study demonstrates that it is hard to find both statistically and clinically significantly associated variables with cognitive impairment in PLWHA, and that the strongest independent association is with depressed mood.

## 1. Background

Human Immunodeficiency Virus (HIV) infection continues to represent a significant public health concern, since, according to epidemiological reports from the end of 2022, it affects over 38 million people worldwide, two-thirds of whom are in Africa. Italy is under the European average with 4.3 new cases/100,000 people per year, more commonly in the age range between 30- and 39-year-old people [1,2,3]. The introduction of new therapies with molecular targets, and, in particular, combination antiretroviral therapy (cART) [4,5] has brought an extremely significant improvement in clinical and immunovirological outcomes [6,7,8], survival [9,10,11], and likely quality of life [12,13]; it has also helped to reduce the incidence of Acquired Immunodeficiency Syndrome (AIDS) [14]. Because the survival of People Living With HIV/AIDS (PLWHA) has greatly increased, they usually reach geriatric age, especially in developed countries, defined as the chronological age of 65 years old or older [3,15,16]. For this reason, PLWHA present with clinical matters that are commonly associated with age, such as cardiovascular, metabolic, and endocrinological diseases, not to mention well-known polypharmacy [17,18,19,20,21,22,23,24]. Another interesting nosological entity is represented by HIV-Associated Neurocognitive Disorder (HAND), a subcortical neurological condition that affects approximately 45% of infected individuals [25]. It is characterised by cognitive problems, such as a striato-frontal profile of impairment, as well as behavioural changes, like apathy and disinhibition. Additionally, motor dysfunction is observed, including fine motor coordination, saccadic eye movement impairment, and gait disturbances [26,27]. During the infection, the involvement of the Central Nervous System (CNS) is affected by the virus, due to its neurotropic nature: indeed, HIV affects both the cerebral cortex and, particularly, subcortical areas [28], such as the amygdala, corpus callosum, and cingulate gyrus [29]. HAND includes different degrees, going from Asymptomatic Neurocognitive Impairment (ANI) and Mild Neurocognitive Disorder (MND) to HIV-1-Associated Dementia (HAD) [30,31,32]. They can be distinguished, according to Antinori criteria [33] by the fact that people with ANI present with performances at least one standard deviation (SD) below the mean in two cognitive domains, people with MND also present with difficulties in carrying out usual daily activities, and people with HAD present with a performances at least two SDs below the mean in two cognitive domains—or at least 2.5 SDs in a single domain, and between 1 and 2 SDs in another domain—in addition to a disability affecting their completion of usual daily activities. The diagnostic and Statistical Manual of Mental Disorders—Fifth Edition (DSM-5) [34] also offers thorough criteria to diagnose various degrees of neurocognitive impairment. These criteria are also supported by neuroimaging techniques, in particular, magnetic resonance imaging, which allows for the identification of cerebral atrophy and ventricular abnormalities [35].

HAND pathophysiology does not seem to involve a direct HIV-mediated neuronal death [36,37], but rather, an overcoming of the blood–brain barrier mediated by infected macrophages [38,39]. Clear roles of astrocyte-derived chemokines [40,41], oxidative stress [42,43], and neuronal apoptosis [44] are also involved. Microglia play a central role in HIV latency, due to their slow cellular division [38] and their resistance to cytopathic effects [45]. Their dysfunction is linked to neurodegenerative diseases [46,47].

Beyond the pathophysiological details, neurocognitive screening tests are employed to assess various domains, including verbal/language abilities, attention/working memory, abstraction/executive function, speed of information processing, and sensory–perceptual and motor skills [48]. However, some tests reliable for the assessment of neurocognitive disorders in the elderly, such as the Mini-Mental State Examination (MMSE) [49,50], did not perform well in recognising HAND [51,52]. MMSE presents two limitations, known as the ceiling and floor effects. The former is observed when the test is administered to individuals with a lower level of education, whereas the latter is encountered when the test is given to those with a higher level of education [53]. This is the reason that led to the validation of more specific screening tools, such as the HIV Dementia Scale (HDS) [54,55] and the International HIV Dementia Scale (IHDS) [56,57], which also present limitations [58,59,60,61]: the two tests did not meet the expected accuracy standards required to provide strong evidence for a diagnosis; therefore, relying solely on these screening tools for diagnostic purposes is not suitable. Among “nonspecific” neurocognitive screening tests, the Repeatable Battery for the Assessment of Neurocognitive Status (RBANS) [62,63] and the Montreal Cognitive Assessment (MoCA) [64,65] have proved useful in HAND assessment. A minor weight is given to immunovirological and other hematic parameters as surrogate markers of cognitive impairment [66,67,68,69], while antiretroviral therapy has shown a questionable role as such [70,71].

This study aims to assess HAND in a PLWHA cohort, in order to find significant associations with cognitive impairment.

## 2. Methods

### 2.1. Design of the Study

A cross-sectional study was conducted in a convenience sample, comprising 210 patients, who were evaluated at the Immunology Service of the University Hospital of Monserrato, located in Cagliari, Italy, between November 2022 and April 2023.

### 2.2. Inclusion/Exclusion Criteria

We included PLWHA on cART aged 18 years or older, who received an immunological evaluation. People undergoing their first immunological evaluation, with missing anamnestic data, or who did not provide informed consent were excluded from the study.

### 2.3. Assessment

The following assessments were conducted among the enrolled subjects to provide a cognitive, affective, and functional evaluation:

○ MoCA [64,65]: It evaluates different cognitive domains: visuospatial and executive abilities, naming, memory, attention, language, abstraction, and spatial and temporal orientation. The first is assessed through a series of exercises, including drawing a line to sequence alternating digits and letters, a cube shape, and a clock showing 10 min past 11. Verbal fluency is tested by identifying the names of three animals. The screening test evaluates short-term and delayed memories by requiring subjects to repeat five words spoken by the examiner immediately and then again after a delay, respectively. Attention is tested through the repetition of a series of numbers forward and backwards, identification of the letter “A” in a sequence of letters, tapping the hand on the table, and a subtraction exercise. Language is assessed by repeating two sentences and producing a list of words starting with the letter “F” within one minute. Abstraction is evaluated by explaining what two pairs of words have in common. Lastly, spatial and temporal orientation are tested by asking the patient about the date and location of the visit. It takes approximately 10 min to administer the MoCA. The test’s total score ranges from 0 (maximum impairment) to 30 (absence of impairment) and is adjusted for education level: an additional point [72] is attributed if the patient has 12 years or less of education. Cognitive impairment is indicated by scores below 26, whereas a normal condition is suggested by scores above 26.
○ Patient Health Questionnaire—two items (PHQ-2) [73,74]: It is a brief screening tool, used to evaluate depressive symptoms and anhedonia over the past two weeks. It consists of two questions, which are the first two items of the PHQ-9. The first question is, “*Over the last two weeks, how often have you been bothered by any of the following problems*?”. The two items under evaluation are “*little interest or pleasure in doing things*” and “*feeling down, depressed, or hopeless*”. Four response options are available, and these are “not at all”, “several days”, “more than half the days”, and “nearly every day”, scored as 0, 1, 2, and 3, respectively. As such, the PHQ-2 score ranges from 0 (no depressive symptoms) to 6 (daily depressive symptoms). A score of 3 or more indicates a depressive mood, with a sensitivity of 83% and a specificity of 90% [73], unlike the PHQ-9 score ≥ 10, whose sensitivity and specificity are 88% [75].
○ Activities of Daily Living (ADLs) [76,77]: The Katz ADL scale is used to indicate a person’s functional status. It evaluates autonomy in common daily routine activities, oriented towards taking care of one’s own body and enabling basic survival and well-being. These activities include personal hygiene, getting dressed, using the toilet, walking, sitting, standing, lying down, climbing stairs, continence, and eating. The scale is scored by assigning 1 if the patient can perform the activity and 0 if he/she cannot. It ranges from 0, indicating complete dependence, to 6, indicating complete autonomy. A score of 3 or 4 indicates moderate impairment, while a score of 2 or less indicates severe functional impairment.
○ Instrumental Activities of Daily Living (IADLs) [76,77]: This assessment evaluates one’s ability to perform daily instrumental activities to support their life at home and in the community. These activities require more complex interactions than those used in ADLs (activities of daily living). Some examples include using the telephone, managing finances, shopping, food preparation, housekeeping, laundry, transportation, and being responsible for taking one’s medications. The score ranges from 0 (complete dependence) to 8 (complete autonomy). A score of 4 or 5 indicates moderate dependence, while a score of 3 or less indicates severe functional impairment.

The patients also underwent blood chemistry tests, such as:

○ Immunovirological assessment: CD4+ lymphocyte count (present and nadir, which means the lowest measured value), CD4+/CD8+ lymphocytes ratio, HIV RNA load levels (present and zenith, which means the highest measured value).
○ Haemoglobin (Hb), white blood cells (WBCs), and platelet (PLT) count, prothrombin time–international normalised ratio (PT–INR), albumin, aspartate aminotransferase (AST), alanine aminotransferase (ALT), creatinine, low-density lipoprotein (LDL), ferritin, vitamin B12, folate, thyroid-stimulating hormone (TSH), C-reactive protein (CRP).

In addition, the assessment was expanded to include the evaluation of antiretroviral therapy, which is categorized into six different pharmacological classes, namely nucleoside reverse transcriptase inhibitors (NRTIs), nucleotide analogues (NtRTIs), integrase inhibitors (INIs), non-nucleoside inhibitors (NNRTIs), protease inhibitors (PIs), and CCR5 inhibitors.

### 2.4. Statistical Analysis

Variables were expressed as medians and interquartile ranges (IQRs) or percentages (%), where appropriate. Multivariate analysis was performed with multiple regression—stepwise (*p*-values > 0.1 were excluded from the model): its results, expressed with their standard errors and odds ratios (ORs), were evaluated through the area under the ROC curve (AUC). In order to apply it, the MoCA was considered with the dependent variable and dichotomization, with 0 indicating the absence of impairment (score ≥ 26) and 1 indicating the presence of impairment (score < 26) (1).

The Chi-squared (χ^2^) test involved a comparison of the singular MoCA score with the abovementioned dichotomization (MoCA ≥ 26 and < 26 points) and the regressor derived from multiple regression. Prior to the application of the test, the regressors were dichotomized using cut-offs of 10 and 15 years for duration of infection, and 60 and 65 years for age.

The results are reported indicating *p*-values, defining a significance level of *p* ≤ 0.05, in reference to 95% confidence intervals (95%CI). MedCalc software (Version 22.005, Ostend, Belgium) was used for the statistical analysis.

## 3. Results

The study included 210 PLWHA, of whom, 137 (65.2%) were males and 25 (11.90%) were geriatric PLWHA, with a median age of 58 years (IQR: 52–61). The characteristics of the enrolled subjects are shown in Table 1 and Table 2. The members of the sample have spent a median of 25 years of their lives with HIV infection, and 97.6% were following a cART regimen: in particular, the most common antiretroviral drugs taken were NRTIs (82.4%), followed by NtRTIs (71.4%), INIs (66.7%), NNRTIs (35.7%), and PIs (15.7%), while only 1.4% were taking CCR5 inhibitors. The immunovirological responses were optimal, as demonstrated by the fact that the median CD4+ lymphocyte count was 769 cells/μL (median nadir: 254 cells/μL), and the median HIV RNA copy number was 0 (median zenith: 58,420 copies/μL).

Cognitive assessment results revealed that a sum of 115 people (54.8%) were found to be cognitively impaired, with a median MoCA score of 25, indicating mild cognitive impairment, observed in 81.74% of patients receiving treatment with an NRTI, 39.13% with an NNRTI, 71.30% with an NtRTI, 17.4% with an IP, 64.35% with an INI, and 1.74% with a CCR5 inhibitor. However, the limit of the third quartile was 27, which indicated normal cognitive function. Regarding the affective assessment, the median PHQ-2 score was 1, indicating a non-depressed mood. However, 50 subjects (23.8%) were found to be depressed. Functional assessment showed that both ADL and IADL independence were preserved among the participants, as demonstrated by the medians (ADLs: 6 out of 6, and IADLs: 8 out of 8), and IQRs (ADLs: 6–6, and IADLs: 8–8). Furthermore, the hematic exams conducted on the participants revealed an overall good control of blood count, and liver, kidney, and thyroid functions. The exams also indicated that the iron, lipid, and vitamin profiles of the participants were satisfactory. Moreover, the absence of systemic inflammation was observed.

In order to evaluate potential factors influencing cognitive performances, we designed a logistic regression, in which MoCA scores were used as the dependent variable, and age, years of HIV infection, PHQ-2, ADLs, IADLs, and the immunovirological and hematic exams shown in Table 1, as well as the different drug classes shown in Table 2, were used as independent variables. As in Table 3, four variables were considered independently associated with MoCA, while the others were excluded from the model (*p* > 0.1): in particular, the duration of infection was negatively associated with cognitive impairment (OR: 0.96, 95%CI: 0.93–0.99, *p* = 0.0099), while age (OR: 1.12, 95%CI: 1.07–1.17, *p* < 0.0001), ALT (OR: 1.02, 95%CI: 1.01–1.04, *p* = 0.0262), and PHQ-2 (OR: 1.33, 95%CI: 1.11–1.60, *p* < 0.0001) were positively associated with cognitive impairment (AUC: 0.725, standard error: 0.03, 95%CI: 0.66–0.78).

**Figure 1:**
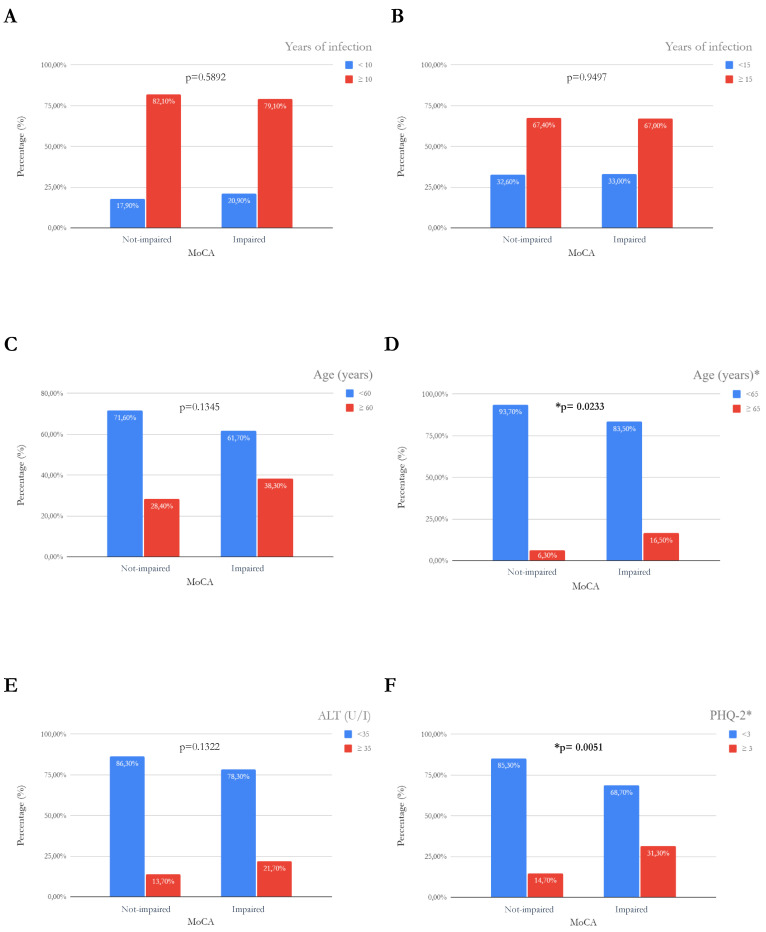
Cognitively impaired vs non-impaired subjects; ALT, Alanine aminotransferase; MoCA, Montreal Cognitive Assessment; PHQ-2, Patient Health Questionnaire—two items. *p*-value refers to blue bars. “*” indicates a significant *p*-value. (**A**) Comparison of subjects (percentages) with normal or deficient MoCA scores, to the duration of infection (less than or equal/greater than 10 years) (**B**) Comparison of subjects (percentages) with normal or deficient MoCA scores, to the duration of infection (less than or equal/greater than 15 years); (**C**) Comparison of subjects (percentages) with normal or deficient MoCA scores, to age (less than or equal/greater than 60 years); (**D**) Comparison of subjects (percentages) with normal or deficient MoCA scores, to age (less than or equal/greater than 65 years); (**E**) Comparison of subjects (percentages) with normal or deficient MoCA scores, to ALT (less than or equal/greater than 35 U/L); (**F**) Comparison of subjects (percentages) with normal or deficient MoCA scores, to PHQ-2 score (less than or equal/above 3).

Subsequently, after performing the χ^2^ test, we found that 20.9% of cognitively impaired and 17.9% of non-impaired people have been infected for less than 10 years (χ^2^: 0.29, *p* = 0.5892), as shown in Figure 1A; meanwhile, 33% of cognitively impaired and 32.6% of non-impaired people have been infected for less than 15 years (χ^2^: 0.004, *p* = 0.9497), as in Figure 1B. Also, we detected that 61.7% of cognitively impaired and 71.6% of non-impaired people were less than 60 years old (χ^2^: 2.24, *p* = 0.1345), as shown in Figure 1C; meanwhile, 83.5% of cognitively impaired and 93.7% of non-impaired people were less than 65 years old (χ^2^: 5.142, *p* = 0.0233), as shown in Figure 1D. We divided ALT using the 35 U/L cut-off: 78.3% of cognitively impaired and 86.3% of non-impaired people had ALT levels lower than 35 U/L (χ^2^: 2.27, *p* = 0.1322), as shown in Figure 1E. Finally, we divided PHQ-2 scores using the three-point cut-off: 68.7% of cognitively impaired and 85.3% of non-impaired people had PHQ-2 scores lower than 3 (χ^2^: 7.834, *p* = 0.0051), as shown in Figure 1F.

## 4. Discussion

The present study aimed to assess HAND with MoCA in a PLWHA cohort, and to find significant associations with cognitive impairment. Our data revealed an overall optimal response to antiretroviral therapy, as evidenced by the excellent levels of CD4+ lymphocytes and HIV RNA copies, as well as the other laboratory values, which extensively explored blood count, kidney, liver, and thyroid functions, iron, lipid, and vitamin assets, and inflammation. Similarly, autonomy in both basic and instrumental daily activities was overall preserved. However, more than 54% of the sample showed cognitive impairment, and we decided to further explore such an aspect by way of a multivariate analysis. The designed stepwise model demonstrated that cognitively impaired people were older, had more depressed moods, and had higher ALT levels; moreover, they had been infected by HIV for a lower number of years.

The results presented are consistent with the existing literature [27,77,78,79] on the association between cognitive impairment and depression in elderly PLWHA. The advent of cART has considerably improved clinical outcomes, leading to an increase in life expectancy for PLWHA. This has further led to a rise in the prevalence of HAND [4,6,9], requiring early detection and management of the condition. While the MMSE has not demonstrated utility as a screening tool for HAND, other tests such as HDS, IHDS, RBANS, and MoCA have shown promise [60,63,64]. However, PLWHA receiving cART generally experience mild cognitive deficits that do not meet the criteria for a dementia diagnosis (HAD), as observed in our study, in which patients presented autonomy in ADLs and IADLs.

Furthermore, HAND is more common in older PLWHA [80], and neurocognitive impairment is often more advanced than in age-matched controls [81]. Additionally, HAND ageing-related comorbidities, such as cardiovascular diseases, kidney disease, neurodegeneration, chronic immune activation, and immune senescence, are likely to impact the neuropathology of HAND, leading to atypical forms of the disease [82]. According to the literature, our findings suggest that HAND is associated with depression, probably due to changes in brain structure [83], somatostatin dysregulation [84], and increased levels of inflammatory cytokines. However, the relationship between HIV and depression is complex and not always consistent. Nonetheless, it has been demonstrated that depressive symptoms are one of the strongest risk factors for cognitive decline [85] and may be correlated with a worse prognosis for PLWHA who are ageing [86].

Regarding the other results, no previous studies have shown a clear association with ALT: in all fairness, the association we found is indeed statistically significant but with an OR of 1.02, which is a clinically insignificant increase. Another inconsistency with the literature—yet a controversial issue—[87] was the inverse relationship between cognitive impairment and duration of HIV infection (OR: 0.96), which is again statistically significant but almost clinically insignificant. In general, all the associations obtained, except for age and depression, failed to show ORs higher than ±10%.

In order to deepen the above, we dichotomized the four independent variables and analysed them separately. As intended, ALT levels and duration of infection lost even their statistical significance when examined individually, as could be foreseen for the abovementioned reasons. Similarly, age was not significant when considering the 60-year threshold. However, when the threshold was raised to 65 years, age became significant, which, not without reason, discriminates adults from elderly people. The association observed in patients aged 65 and above, who exhibit cognitive impairment, can be attributed to two factors. Firstly, the neurodegenerative damage that is mediated by HIV and, secondly, the age-related mechanisms that are implicated in the pathogenesis of cognitive impairment.

The other significant association was, again unsurprisingly, with depression, as a large portion of depressed people are also cognitively impaired.

However, it is important to acknowledge that the present study has certain limitations. Notably, the immunovirological status of patients concerning Hepatitis B Virus (HBV) and Hepatitis C Virus (HCV) has not been included as a quantitative variable in the statistical analysis. This is mainly due to the unavailability of recent test results and/or information regarding the patient’s history of HCV eradication therapy or HBV vaccination. Additionally, the limited amount of time dedicated to the administration of the screening test did not allow for a detailed anamnesis regarding patients’ habits, such as the use of alcohol or narcotics. Furthermore, the assessment of the patient’s affective state was limited to the PHQ-2, without administering the PHQ-9 to patients who showed suggestive scores for depressive symptoms. Finally, the type of study conducted may limit the generalizability of the results obtained. Therefore, it is recommended that future research be conducted as a longitudinal study, with the implementation of an anamnestic evaluation of voluntary habits, laboratory evaluation of the serological status for HBV and HCV, and affective evaluation using the PHQ-9, in order to allow for a comparison of the sensitivity and specificity of the PHQ-2 and PHQ-9 tests.

## 5. Conclusions

Our study demonstrates that it is hard to find both statistically and clinically significantly associated variables with cognitive impairment in PLWHA, even considering an extensive hematic panel and the assessment of mood and functional autonomy. It also demonstrates that the strongest independent association is with depressed mood, the assessment of which cannot and should not be avoided, since it can significantly affect cognitive performances in younger and older PLWHA.

## Figures and Tables

**Table 1:** Characteristics of the sample.

Variable	Minimum	Maximum	Median	IQR
Age (years)	30	81	58	52–61
Years of HIV infection	0	40	25	13–31
MoCA (adjusted for ages of school)	9	30	25	23–27
PHQ-2	0	6	1	0–2
ADLs	3	6	6	6–6
IADLs	3	8	8	8–8
CD4+ T-cells (cells/μL)	79	1978	769	540–1003
CD4+/CD8+ T-cell ratio	0.09	11.12	1.04	0.66–1.53
CD4+ T-cell nadir (cells/μL)	3	804	254	118–392
HIV RNA (copies/μL)	0	215	0	0–0
HIV RNA zenith (copies/μL)	20	17,270,926	58,420	17,100–161,000
Hb (g/dL)	8.7	19.5	14.6	13.3–15.7
WBCs (×10^3^/μL)	2.27	12.72	6.16	5.11–7.28
PLTs (×10^3^/μL)	52	451	224	177–264
PT (INR)	0.86	6.61	0.95	0.93–0.98
Albumin (g/dL)	3.45	5.09	4.3	4.07–4.48
AST (U/L)	9	196	21	18–27
ALT (U/L)	9	143	20	14–29
Creatinine (mg/dL)	0.73	6.02	1.05	0.93–1.19
LDL (mg/dL)	16	216	111	88–130
Ferritin (μg/L)	4.4	1133.2	105.8	59–178.7
Vitamin B12 (ng/mL)	237	1225	495.5	406–598
Folate (ng/mL)	1.7	24	9.5	7–13.4
TSH (μU/L)	0.05	16.57	1.59	1.1–2.12
CRP (mg/L)	0	65.3	1.1	0.4–4

ADLs, Activities of Daily Living; ALT, Alanine aminotransferase; AST, Aspartate aminotransferase; CRP, C-reactive protein; Hb, Haemoglobin; HIV, Human Immunodeficiency Virus; IADLs, Instrumental Activities of Daily Living; IQR, Interquartile range; LDL, Low-density lipoprotein; MoCA, Montreal Cognitive Assessment; PHQ-2, Patient Health Questionnaire—two items; PLTs, Platelets; PT–INR, Prothrombin time–international normalized ratio; TSH, Thyroid-stimulating hormone; WBCs, White blood cells.

**Table 2:** Antiretroviral therapy.

Variable	%
Nucleoside reverse transcriptase inhibitor (NRTI)	82.4
Nonnucleoside reverse transcriptase inhibitor (NNRTI)	35.7
Nucleotide reverse transcriptase inhibitor (NtRTI)	71.4
Protease inhibitor (PI)	15.7
Integrase inhibitor (INI)	66.7
CCR5 inhibitor	1.4

**Table 3:** Logistic regression—stepwise (y = MoCA).

Variable *	Standard Error	OR	95%CI	*p*
Years of infection	0.017	0.96	0.93–0.99	0.0099
Age (years)	0.023	1.12	1.07–1.17	<0.0001
ALT (U/L)	0.009	1.02	1.01–1.04	0.0262
PHQ-2	0.094	1.33	1.11–1.60	<0.0001

* *p* > 0.1 excluded from the model ALT, Alanine aminotransferase; CI, Confidence interval; MoCA, Montreal Cognitive Assessment; OR, Odds ratio; PHQ-2, Patient Health Questionnaire—two items.

## Data Availability

The data and materials used and/or analysed during the current study are not publicly available. They are available from the corresponding author upon reasonable request.

## Abbreviations

Acquired Immunodeficiency Syndrome (AIDS); Activities of Daily Living (ADLs); Alanine aminotransferase (ALT); Area under the ROC curve (AUC); Aspartate aminotransferase (AST); Asymptomatic Neurocognitive Impairment (ANI); C-reactive protein (CRP); Diagnostic and Statistical Manual of Mental Disorders—Fifth Edition (DSM-5); Combination antiretroviral therapy (cART); Central Nervous System (CNS); HIV-1-Associated Dementia (HAD); HIV-Associated Neurocognitive Disorder (HAND); HIV Dementia Scale (HDS); Haemoglobin (Hb); Hepatitis B Virus (HBV) Hepatitis C Virus (HCV), Human Immunodeficiency Virus (HIV); Instrumental Activities of Daily Living (IADLs); International HIV Dementia Scale (IHDS); Interquartile range (IQR); Low-density lipoprotein (LDL); Mild Neurocognitive Disorder (MND); Mini-Mental State Examination (MMSE); Montreal Cognitive Assessment (MoCA); Nonnucleoside Reverse Transcriptase Inhibitors (NNRTIs); Nucleoside Reverse Transcriptase Inhibitors (NRTIs); Nucleotide Reverse Transcriptase Inhibitors (NtRTIs); Odds Ratio (OR); Patient Health Questionnaire—two items (PHQ-2); People living with HIV/AIDS (PLWHA); Platelets (PLTs); Protease Inhibitors (PIs); Prothrombin time–international normalized ratio (PT–INR); Repeatable Battery for the Assessment of Neurocognitive Status (RBANS); Standard deviation (SD); Thyroid-stimulating hormone (TSH); White blood cells (WBCs)
